# Accuracy of portable artificial intelligence-supported fundus camera in the screening of diabetic retinopathy in primary care

**DOI:** 10.20945/2359-4292-2026-0051

**Published:** 2026-05-15

**Authors:** Daniela O. Alves, Daniel Lavinsky, Myriam E. B. Strzalkowski, Leila B. Moreira

**Affiliations:** 1 Programa de Pós-graduação, Departamento de Educação, Hospital de Clínicas de Porto Alegre, Porto Alegre, RS, Brasil; 2 Departamento de Oftalmologia, Hospital de Clínicas de Porto Alegre, Porto Alegre, RS, Brasil; 3 Escola de Enfermagem, Universidade Federal do Rio Grande do Sul, Porto Alegre, RS, Brasil

**Keywords:** Diabetes mellitus, diabetic retinopathy, artificial intelligence, screening, primary health care

## Abstract

**Objective:**

Research on the use of portable fundus cameras utilizing artificial intelligence (AI) for diabetic retinopathy (DR) screening in primary care remains limited. We aimed to evaluate the accuracy and reliability of DR screening in primary care using a smartphone-based, AI-assisted device in a small municipality in southern Brazil.

**Materials and methods:**

The reference standard was classification of fundus images by a retina specialist. Patients with diabetes enrolled in the Brazilian Family Health Program were recruited for the study. A general ophthalmologist obtained fundus images from 134 patients, and a retina specialist validated the DR diagnosis by AI.

**Results:**

The sample was predominantly female, with most patients having type 2 diabetes mellitus (T2DM). The age ranged from 17 to 81 years. Blood pressure was controlled in 34.9% of the sample. HbA1c levels ranged from 5.4% to 13.9%, and 35.3% of participants had levels below 7.0%. After excluding eight participants due to low image quality, the DR prevalence was 24.6%. The AI-based screening test for DR in primary care demonstrated a sensitivity of 100% (95% CI 88.8-100) and a specificity of 66.3% (95% CI 55.9-75.7). The negative predictive value (NPV) was 100% (95% CI 94.3-100), and the positive predictive value (PPV) was 49.2% (95% CI 36.4-62.1).

**Conclusion:**

The smartphone-based, AI-assisted device showed good accuracy and excellent performance for DR screening in primary care. It can avoid unnecessary medical referrals and help prioritize patients with advanced disease who require early treatment to prevent severe complications.

## INTRODUCTION

In 2021, type 2 diabetes mellitus (T2DM) accounted for 75.3 million (95% UI 63.5-90.2) global disability-adjusted life years (DALYs) ^(1)^, representing 2.6% (2.3-2.9) of total global DALYs. T2DM contributed to 95.4% of the total diabetes-related burden. Since 2010, the percentage increase in DALYs attributable to T2DM was 52.2%. The International Diabetic Federation ^(2)^ estimated that 996 billion U.S. dollars were spent on diabetes care, and that three of every four adults with diabetes live in low-income countries.

Diabetic retinopathy (DR) is one of the leading chronic complications of diabetes mellitus (DM), affecting about one-third of patients. Among them, 10% are at risk of irreversible vision loss, and some may progress to blindness ^(3)^. DR is the leading cause of vision loss among working-age adults and a threat to the quality of life for millions worldwide ^(3-5)^, mainly because it is asymptomatic in early stages. Many studies ^(6-8)^ have demonstrated that DR screening is cost-effective in preventing irreversible vision loss and morbidity. However, implementing screening programs remains challenging in large countries with unequal access to healthcare services, especially in low-income regions ^(9)^. Therefore, cost-effective alternatives to in-person ophthalmologist evaluations or seven-field color fundus imaging have been explored ^(10)^. These methods include portable fundus cameras, artificial intelligence (AI), and telemedicine.

Studies have demonstrated positive results for both AI-only models and portable retinal cameras ^(11-14)^. However, research on the use of portable fundus imaging combined with AI for DR screening in primary care remains limited. In India, two studies ^(15,16)^ validated the reliability of a portable retinal camera equipped with offline AI software, operated by non-physician healthcare professionals. Our study aims to evaluate the accuracy and reliability of an AI-assisted, smartphone-based device for DR screening in primary care in a small municipality in southern Brazil.

## MATERIALS AND METHODS

This cross-sectional observational study, prospectively designed, was conducted to screen for retina changes in individuals with diabetes using a smartphone-based portable retinal camera combined with AI software in a primary care center in the city of Glorinha - State of Rio Grande do Sul - in southern Brazil. The municipality has about 8,000 inhabitants and 733 patients with diabetes registered in the municipal pharmacy. It has broad coverage by the Brazilian Unified Health System (*Sistema Único de Saude* - SUS) via family health services and a large percentage of rural residents.

Patients with diabetes enrolled in the Family Health Program were recruited via various methods: direct contact during routine visits to healthcare units, active outreach by health agents and scheduled medical appointments, direct engagement during *HIPERDIA* group activities (a Ministry of Health program for patients with arterial hypertension and diabetes), and advertisements via social media platforms (Instagram and Facebook) or posters displayed in local health units. All participants received detailed information about the study and provided written informed consent. The study adhered to the principles of the Declaration of Helsinki. The Institutional Ethics and Research Committee approved the project (CAAE 67676323.0.000.5327).

**Figure S1:**
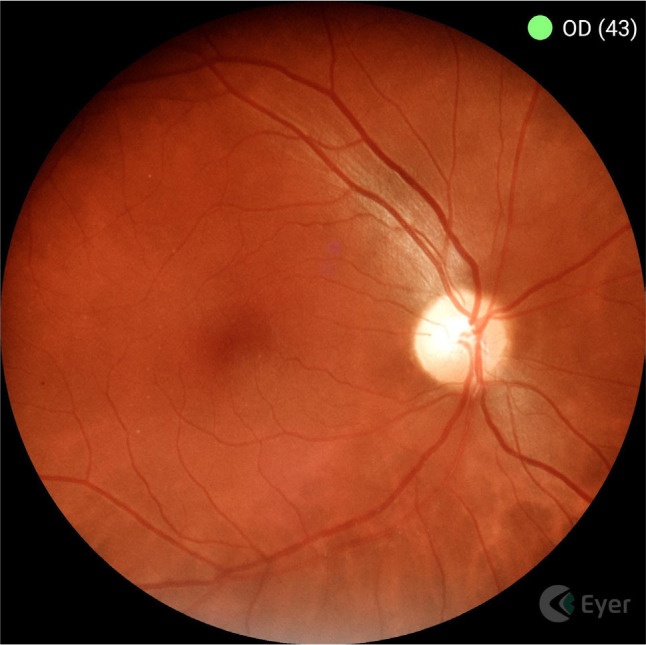
Absence of DR (AI shows green circle).

**Figure S2:**
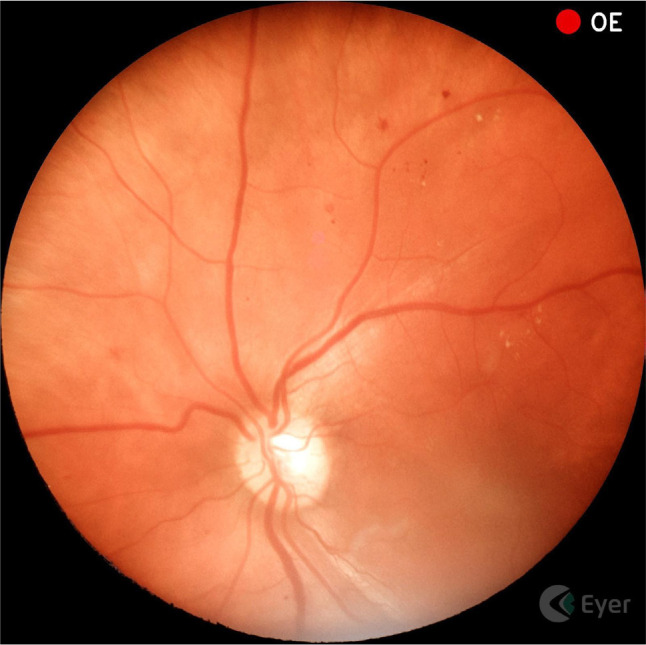
Moderate NPDR; AI shows a red circle, indicating the presence of a change.

**Figure S3:**
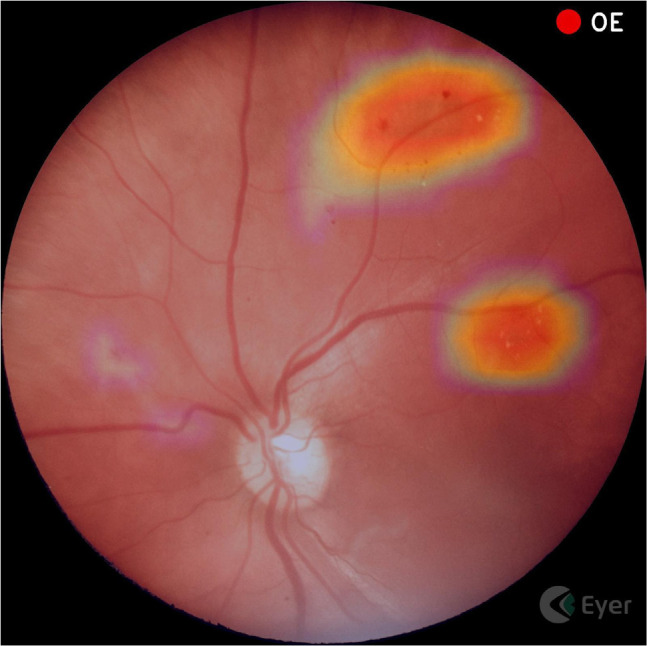
Moderate NPDR (the same **Figure S2**) with AI markings.

**Figure S4:**
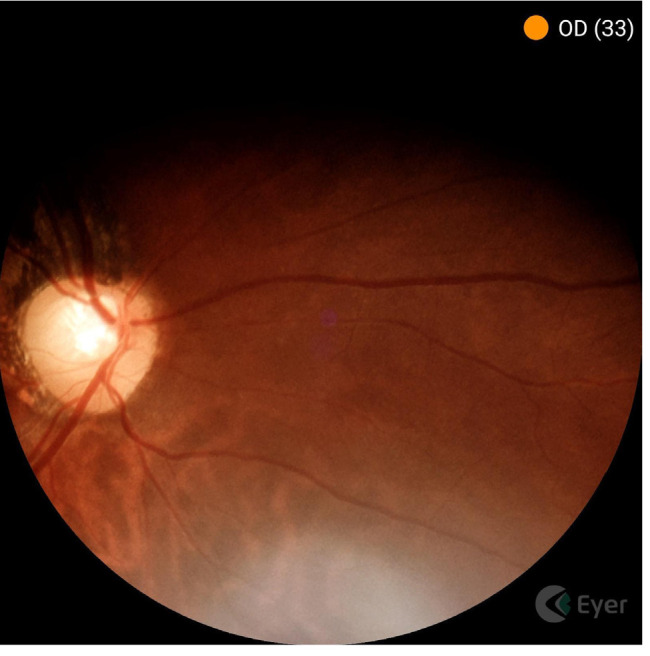
Fundoscopy change that is not DR, identified by AI (orange circle). Optic nerve with suspected glaucoma, and need further evaluation.

**Figure S5:**
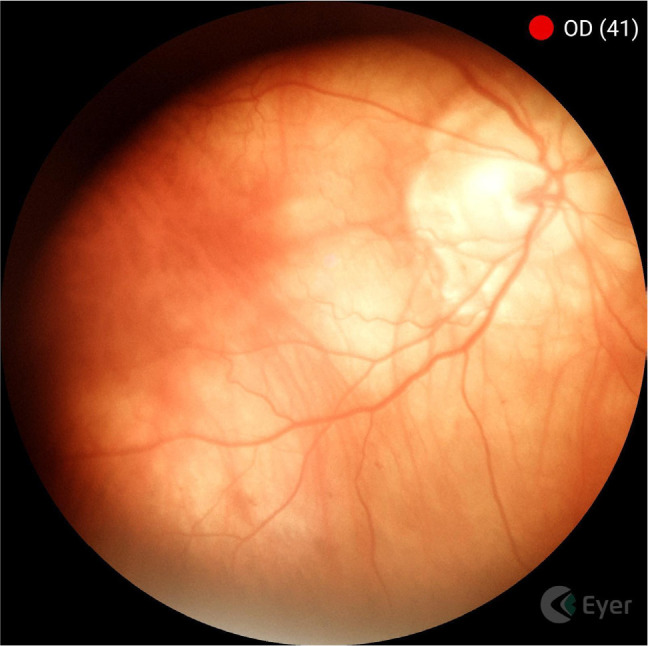
Fundus change detected by AI, but not DR.

**Figure S6:**
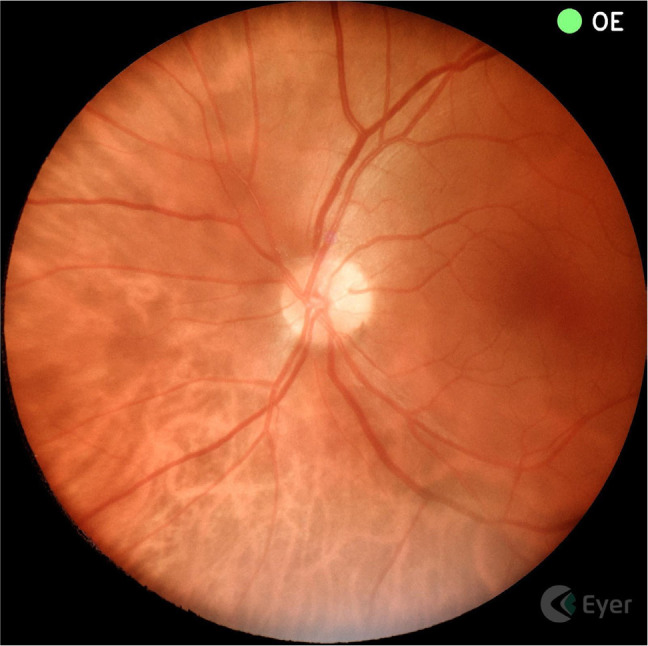
Non-pathological retinal change; AI does not identify a problem.

**Figure S7:**
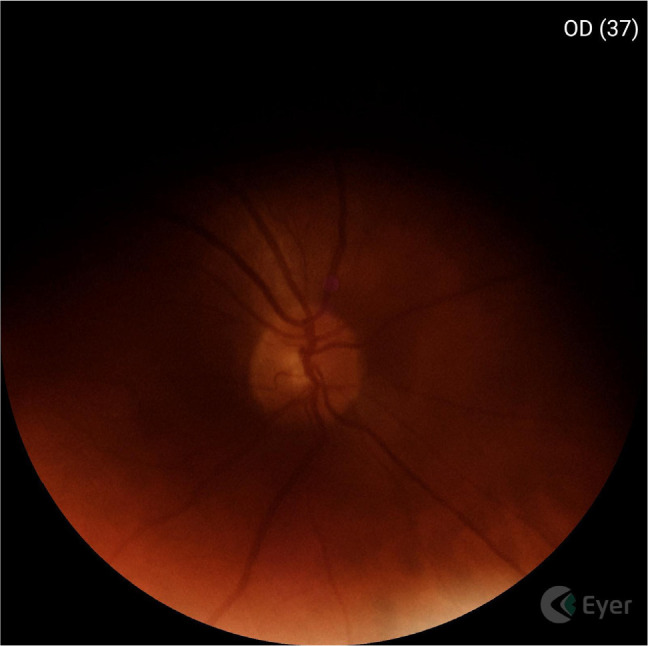
Fundus image impossible to classification; AI does not show any color.

We used a smartphone-based handheld device (Eyer^®^, Phelcom Technologies - São Carlos, Brazil) connected to EyerCloud^®^, a cloud storage system. Mydriasis is not mandatory when using this camera. Images were analyzed in real-time by a general ophthalmologist and by EyerMaps^®^, an AI software that detects retinal changes and classifies the images by displaying a color-coded indicator in the upper right corner: green (low probability of alteration), yellow (medium probability of alteration), and orange or red (high probability of alteration). If the image quality is insufficient for AI analysis, no color indicator is displayed. Fundus imaging examples are shown in the supporting file (**Figures S1-S7**). The reference standard was the analyses and classification of fundus images by a retina specialist, as patients with diabetes in primary care in Brazil are typically referred to specialists for DR screening. The AI software is a direct resource of the fundus camera and does its analysis automatically when the image is taken. It does not use any information other than the image captured to make the analysis. The fundus camera device and the AI model were developed in Brazil using local data mostly (some contribution from North America and European countries), and are approved by ANVISA, the Brazilian regulatory agency. Both were validated in the Brazilian population ^(14,17)^.

Color fundus imaging was performed by a general ophthalmologist. Fundus images obtained were registered in medical records at the health unit and informed to patients. The diagnosis and classification of DR were posteriorly made by a retina specialist, according to the International Classification of Diabetic Retinopathy criteria ^(18)^: 1 - no DR, 2 - mild non-proliferative DR (NPDR), 3 - moderate NPDR, 4 - severe NPDR, 5 - proliferative DR (PDR), 6 - post-laser treatment, 7 - inconclusive. When images were of insufficient quality for AI analysis, no color indicator appeared in the upper right corner; these cases were categorized as negative AI results for the purpose of evaluating test performance. The retina specialist was blinded to the patients’ demographic and clinical data, having access only to images stored as PDF files identified by a numeric code attributed to each patient at the time of evaluation. The images in those PDF files included the AI-attributed color code. The retina specialist was clearly instructed to evaluate images only for DR changes. The fundus classification was registered in a spreadsheet, and results were transcribed and verified using Research Electronic Data Capture (REDCap) ^(19)^. We followed established clinical guidelines ^(20)^ to classify patients as either “non-referral” or “referral” cases for DR: patients with no DR or mild NPDR (non-referral) were advised to continue monitoring clinical parameters in primary care with a follow-up fundus exam in 12 months, while those with moderate NPDR or more severe DR (referral) were referred to a specialist in secondary or tertiary level. Additionally, the general ophthalmologist performing the fundus imagens could identify abnormalities corresponding to DR or other conditions, as well as referring patients when necessary. We defined metabolic control as glycated hemoglobin (HbA1c) below 7.0% ^(21)^, and controlled blood pressure was defined as below 130/80 mmHg ^(22)^, according to current recommendations.

Participants were consecutively included according to the inclusion criterion, which was having a diagnosis of DM. The exclusion criterion was having any condition that prevented fundus imaging, such as lack of patient cooperation or severe media opacities. To perform the exam, 1% tropicamide could be instilled in both eyes to optimize data collection, provided there were no contraindications. While waiting for mydriasis, demographic and clinic data were obtained via interviews and health unit’s records and inserted into the database. Subsequently, each patient was registered in the EyerCloud^®^ system, and at least two images were captured - one centered on the macula and another on the optic nerve. If necessary, new images were taken to obtain two good quality images. When possible, a retina map was obtained using five or more images to include the mid-peripheral retina.

A sample size of 139 subjects was calculated to estimate the proportion of DR cases, with a 10% confidence interval width. Accounting for a 10% potential loss, the target sample size was increased to 155. The calculation (Wald method) considered a 95% confidence level and an expected DR prevalence of 10%, and was calculated using the online PSS Health tool ^(23)^.

Descriptive statistics (means and proportions) were used to characterize the sample. DR prevalence was presented as a rate with a 95% confidence interval. The association between DR and potential risk factors or outcomes was analyzed using the chi-squared test. Agreement between the retina specialist and the AI system was evaluated using Cohen’s kappa coefficient. Test sensitivity, specificity, and positive and negative predictive values with a 95% confidence interval were calculated.

## RESULTS

**Figure 1:**
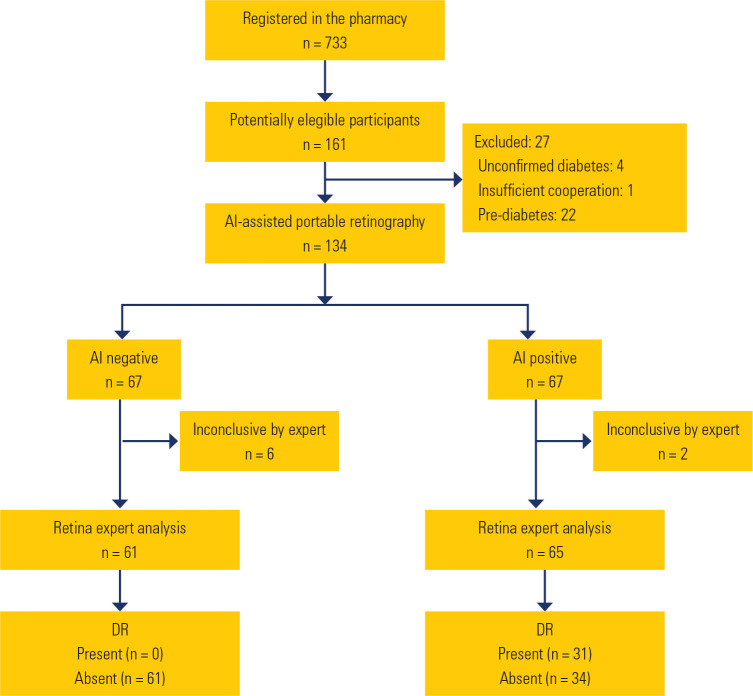
Study flowchart

We collected data from May 17, 2023, to November 25, 2023, from 130 participants and from another 26 participants from July 05, 2025, to August 02, 2025. We excluded 4 participants as they failed to confirm a DM diagnosis in their medical records, 22 pre-DM, and one participant was excluded due to an inability to cooperate during the exam. To perform the exam, 36 patients did not receive mydriatic eye drops because they had to leave the health facility by driving a vehicle after the exam, or there was no need for mydriasis. Fifty two percent of participants evaluated had their first recommended fundus examination after being diagnosed DM. Of the 134 subjects evaluated, 126 were included in the accuracy analysis (**Figure 1**).

**Table 1** shows the characteristics of the total sample (n = 134) and of the subgroup analyzed for DR presence or absence (n = 126), according to the retina specialist’s interpretation. The sample was predominantly composed of women and individuals with T2DM, and most participants had not undergone a fundus examination since their diagnosis. Age ranged from 17 to 81 years, and time since diagnosis varied from less than one year to 42 years. From all sample (n = 134), 54 participants (40,1%) had a body mass index greater than 30 kg/m^2^. Blood pressure was controlled in 34.9% of the sample. Among the 134 participants, 77 reported symptoms in the lower limbs (e.g., pain or discomfort), with the most common (55%) being fatigue, cramps, or itching. These were most frequently located in the calves and tended to be more intense at night. HbA1c values ranged from 5.4% to 13.9% (available for 116 participants), and 35.3% had HbA1c levels < 7.0 mg/dL. Participants with DR had higher systolic blood pressure, longer duration of illness, and a higher prevalence of current or previous diabetic ulcers and presumed peripheral neuropathy.

**Table 1:** Demographic description and clinical characteristics in the sample by the presence of DR (average and SD or n and %)

	Sample (n = 134)	DR positive (n=31)	DR negative (n=95)	p
Female	73 (54.5)	18 (58.1)	51 (53.7)	0.67
Age, years	62.5 (±13)	61.4 (±11.1)	63.1 (±13.0)	0.51
HbA1c (%)	8.3 (±2.1)	9.1 (±2.23)	8.0 (1.9)	0.01
Systolic BP (mmHg)	131.9 (±17.1)	138.9 (±18.5)	129.7 (±16.4)	0.01
DiastolicBP (mmHg)	77.3 (±10.6)	79.5 (±12.5)	76.6 (±10.1)	0.02
BMI (kg/m^2)^	29.5 (±5.2)	29.9 (±5.2)	29.0 (±4.8)	0.379
DM type				0.64^*^
DM1	6 (4.5)	2 (6.5)	4 (4.2)	
DM2	128 (95.5)	29 (93.5)	91 (95.8)	
Hypertension	111 (82.8)	27 (87.1)	77 (81.1)	0.44
Dyslipidemia	96 (71.6)	21 (67.7)	68 (71.6)	0.68
Previous stroke	10 (7.5)	4 (13.3)	6 (6.3)	0.25^*^
Previous heart attack	22 (16.5)	4 (13.3)	17 (17.9)	0.56
Hypothyroidism	32 (23.9)	4 (12.9)	27 (28.4)	0.08
Diabetic ulcer	30 (22.4)	9 (29.0)	20 (21.1)	0.36
Renal dysfunction	21 (15.7)	3 (9.7)	17 (17.9)	0.40^*^
NSS≥3	66 (49.3)	19 (61.3)	43 (45.3)	0.12

BMI: body mass index; NSS: Neuropathy Symptom Score; DR: diabetic retinopathy

* Fisher exact test

The sensitivity of portable retinography with AI as a screening tool for DR in primary care was 100% (95% CI 88.8-100), while specificity was 66.3% (95% CI 55.9-75.7) (**Table 2**). The negative predictive value (NPV) was 100% (95% CI 94.3-100), and the positive predictive value (PPV) was 49.2% (95% CI 36.4-62.1). The agreement between AI and the retina specialist, measured by the Kappa coefficient, was 0.492 (p-value < 0.001).

**Table 2:** Accuracy of portable retinography using artificial intelligence (AI) versus retina specialist assessment for diagnosing diabetic retinopathy (n=126)

	Retina specialist positive	Retina specialist negative	TOTAL
AI positive	31	32	63
AI negative	0	63	63
TOTAL	31	95	126

p <0.001; kappa = 0.492.

Excluding the eight participants whose images were deemed inconclusive by the retina expert, the prevalence of DR diagnosed by the specialist was 24,6% (CI 95% 17.4-33.1). Among the 31 participants with DR, 45.2% (n = 14) had mild NPDR, 45.2% (n = 14) had moderate NPDR, 3,2% (n = 1) had PDR and 6.4% (n = 2) had undergone laser treatment. No cases of severe NPDR were identified.

An exploratory analysis excluding all three patients hose images had no sufficient quality for IA classification showed Sensitivity 100%, Specificity 63%, PPV 48%, NPV 100%.

The results of the AI-based test and the reference standard (retina specialist evaluation) are presented in **Table 3**. The AI algorithm indicated abnormal findings in the presence of moderate NPDR or worse, choroidal nevus, optic nerve changes suggestive of glaucoma, drusen, and other nonspecific or non-pathological alterations. Out of the 67 patients (**Figure 1**) AI had not indicated funduscopic changes, six were inconclusive by retina specialist and 61 showed no signs of DR by retina specialist evaluation (resulting in no false negatives). In the group of ungradable images - for both AI and specialist - cataracts were the main reason for the inability to classify the images. All patients with significant media opacity were referred to further evaluation. The researcher, however, could not classify the cataracts.

**Table 3:** Results of the artificial intelligence (AI) test by the results of the reference standard (N = 134)

Reference standard	AI + n (%)	AI - n (%)	Total
Inconclusive	2 (3.1)	6 (8.7)	8 (6.0)
No apparent DR	32(49.2)	63 (91.3)	95 (70.9)
Mild NPDR	14 (21.5)	0	14 (10.4)
Moderate NPDR	14 (21.5)	0	14 (11.2)
PRD	1(1.5)	0	1 (0.7)
Post-laser	2 (3.1)	0	2 (1.5)
Total	65 (48.5)	69 (51.5)	134

DR: diabetic retinopathy; NPDR: non-proliferative DR; PDR: proliferative DR.

Among the 126 participants whose fundus images could be graded by the retina specialist, and according to the dichotomous classification based on clinical referral, 109 (86.5%) were classified as “non-referrable” DR - meaning they could remain under primary care follow-up, with a new exam recommended in 12 months. In contrast, 17 participants (13.5%) presented “referrable” DR, requiring evaluation and treatment at a higher level of care. In this subgroup, the EyerMaps^®^ software did not fail to detect changes in any of the cases. In this context, the sensitivity and negative predictive value remained at 100%, while the specificity was 57.8% and the positive predictive value was 27.0%. Ten individuals were classified as “inconclusive” by the specialist, being eight negative and two positive for any alteration by AI, and were excluded from the test attributes analysis.

## DISCUSSION

In this cross-sectional study with 126 patients with DM, we aimed to evaluate the performance of an AI-supported portable fundus camera for DR screening in primary care. According to the specialist’s classification of fundus imaging, the prevalence of DR was 24.6%. The AI-supported portable fundus camera demonstrated a sensitivity of 100% and a specificity of 66.3% for DR detection. The NPV was 100%, while the PPV was 49.2%. These findings corroborate the impression already obtained by other studies that AI-assisted portable retinography is an excellent screening tool ^(13,14,16)^, as it has high specificity and high NPV. This feature means the test is effective and safe to be implemented as a screening test in primary care.

The prevalence of microvascular complications - such as any form of DR - found in the primary care diabetic patient sample is among the lowest reported in studies included in a brazilian meta-analysis ^(24)^. The summarized prevalence in the meta-analysis was 33% and prevalence ranged between studies from 5.3% to 85.7%. A study ^(13)^ aiming DR screening conducted in a Brazilian urban primary care setting with a handheld smartphone-based retinal camera recorded DR prevalence of 24.1% in a sample of 439 individuals with DM. The rate is included in the CI95% of our study. The prevalence rates vary widely across studies due to differences in study design, populations, sample characteristics, including age and DM duration. The most prevalent risk factors for DR observed in this study align with previous findings: DM duration, age over 50 years, comorbidities such as hypertension, overweight or obesity, heart attack and stroke history, and insufficient metabolic control ^(3,13,25)^.

Even in countries with established national protocols for screening DM complications, coverage remains challenging ^(26-28)^. Notably, about 50% of patients evaluated had their first recommended fundus examination after being diagnosed with DM in this study. In Brazil, most of the population is assisted by SUS ^(29)^, and according to the Ministry of Health recommendations ^(30)^, patients diagnosed with DM in primary care should be referred to an ophthalmologist for fundoscopy every 12 months. Specialists are typically available only at secondary or tertiary care levels, resulting in several months of waiting for ophthalmologist consultation. AI-supported smartphone-based portable retinography may be a solution to improve DM assistance.

AI programs have been developed and validated for ocular disease screening, including DR ^(11-13,15,16,31)^. Most algorithms are programmed to detect significant changes in DR, such as hard exudates, flame-shaped hemorrhages, and significant vascular abnormalities. Some algorithms can identify patients with DR who require referral to a retina specialist (“referrable DR”). Others can differentiate between normal fundus imaging and those showing possible pathologic changes, as well as indicate when image quality is insufficient for analysis.

In Mumbai, India, between 2018 and 2019, a study ^(16)^ involving 1,378 primary care patients evaluated the reliability of an offline AI in a smartphone-based fundus camera for community screening of DR by healthcare workers. Like us, they included rural population, although ethnically different from that of Brazil. Images were captured using the smartphone-based portable fundus camera Remidio Non-Mydriatic Fundus on Phone (Remidio Innovative Solutions Pvt. Ltd.). Patients were diagnosed as referable DR and non-referable DR by the algorithm based on characteristic DR images. Differences between algorithms may explain the higher specificity than our study. The sensitivity was 100%, with specificity close to 90%. For any DR, sensitivity was 89.13% and specificity was 94.43%, based on assessments by two blinded retina specialists. Our study showed high sensitivity to identify any abnormalities in the fundus image - not limited to DR - which explains the higher number of false positives when considering only DR. The high NPV indicates that if no abnormalities are detected, the patient is unlikely to have DR. Therefore, this AI-supported camera enables effective screening to identify patients who require additional evaluation and intervention at specialized centers. To enhance specificity in the method used in our study regarding RD, telemedicine consultation can be associated.

Furthermore, AI software is highly effective in selecting patients who can safely continue follow-up in primary care with another exam scheduled after one year. Consequently, AI-supported portable retinography may be a cost-effective method for DR screening in primary care. Souza and cols. ^(32)^ assessed applicability and economic viability of fundus photography-based teleophthalmology screening for DR in two referral health centers from different cities in the state of Minas Gerais-BR comparing its cost with that of an ordinary ophthalmology visit. They concluded that the method was a viable, effective, and significantly cheaper strategy for the screening of DR, with cost reduction of 28.76 US$ per patient.

A study conducted in China ^(33)^ assessed the cost-effectiveness of AI-based and ophthalmologist-led DR screening in rural populations using a Markov model for health economic assessments. From the health system perspective, compared to no screening, AI-based screening incurred an incremental cost was approximately USD 5,211.31 with an incremental utility of 0.33. Compared to AI-based screening, ophthalmologist-led screening had an incremental cost of approximately USD 2,070.19 but resulted in a negative incremental utility of -0.31. Compared to no screening, the incremental cost-effectiveness ratio (ICER) for AI-based DR screening was USD 15,595.47 per quality-adjusted life year (QALY), which is less than the ICER threshold in China of USD 30,224.03. A study ^(34)^ conducted in Ontario suggests that a portable retinal camera is a more cost-effective means of screening for DR than a retina specialist in isolated First Nations communities. It is possible that the RD screening method with portable retinography and AI will also be cost-effective for the Brazilian public health system to prevent blindness.

The AI algorithm integrated with the smartphone-based portable fundus camera used in our study can identify and grade several retinal alterations. It cannot diagnose the underlying disease or condition leading to false positive results regarding DR, but it is effective in providing proper health care for those individuals needing specialist evaluation. Due to the method’s low specificity, patients with false-positive DR results will be referred to specialists for other, not necessarily pathological, changes, which may increase the demand for ophthalmological appointments. On the other hand, the high NPV prevents unnecessary referrals for DR screening. The next step to perform with Brazilian data is assessing cost-effectiveness of AI-assisted portable retinography and the financial impacts of wide implementing this tool to the primary care assistance.

Our study has some limitations. The sample size was calculated for a prevalence of DR lower than that recorded in the study, resulting in wide confidence interval for the estimated DR prevalence. Some clinical data were collected retrospectively from patients’ records, with missing data in some variables. On the other hand, the missing data do not bias the main results of the study. The retina specialist received subjects’ images containing the color code provided by the AI software. Although the specialist was aware of how the EyerMaps^®^ software works and was instructed to analyse the fundus images only for DR, the color code might unconsciously have influenced the specialist analysis. It can be a potential bias that may have increased the DR prevalence, and influenced sensitivity and specificity rates. Although the rates should be seen with caution, they are in consonance with similar studies ^(13,32)^. Finally, the study was conducted at a single center, limiting the generalization of the results to the general population of diabetics assisted in the primary care of SUS in Brazil.

The portable AI-based fundus camera used in our study is user-friendly and efficient, enabling image capture and AI-based diagnosis. It can be operated by non-medical professionals or generalist practitioners in primary care units, provided they receive adequate training in fundus photography ^(35)^. Equipped with wireless internet connectivity, the device can synchronize all data to the cloud, enabling telemedicine applications. This image storage facilitates continued data updates and patient follow-up, data sharing between primary care teams and other centers, and supports retrospective research. It is essential to highlight ethical issues that must be evaluated and managed cautiously.

In conclusion, AI-supported portable fundus cameras have emerged as a disruptive tool for DR screening, diabetes management and optimizing medical referral processes. This technology can reduce unnecessary medical referrals while ensuring timely and precise identification of severe cases at risk of significant complications if left untreated. Furthermore, AI-assisted portable retinography can offer improved DR screening worldwide, reducing costs, waiting time, and DM-related morbidity.

## Figures and Tables

Healthy optic nerve, blood vessels without changes, no hemorrhages of exudates, good macula visualization.

Optic nerve with normal appearance. Presence of microaneurysms and microhemorrhages (small red dots) in at least three quadrants, as well as the presence of some hard exudates (round yellow lesions near vessels).

Same image as above with AI markings in the anterior “heat map,” highlighting the areas where abnormalities were identified.

Enlargement of the optic nerve cupping and peripapillary atrophy.

Myoptic choroidopathy, a condition present in subjects with high grade myopia (at least 6 spherical diopters). This patient has low vision and risk of retinal detachment.

White stretch marks represents atrophy of the retinal pigment epithelium: variation of the normality and does not need additional evaluation.

Image without quality to evaluate presence or absence of DR changes.

## Data Availability

datasets related to this article will be avail-able upon request to the corresponding author.

## SUPPLEMENTARY MATERIALS

Images examples of retinography captured in the study.
